# Comment on Naranjo-Bonilla et al. Retinal and Choroidal Effects of Continuous Positive Airway Pressure as Treatment for Sleep Apnea: Results at 12 Months. *Int. J. Environ. Res. Public Health* 2022, *19*, 12637

**DOI:** 10.3390/ijerph20021139

**Published:** 2023-01-09

**Authors:** Aniello La Marca, Danilo Biondino, Marco Gioia

**Affiliations:** Department of Medicine, Surgery and Dentistry “Scuola Medica Salernitana”, University of Salerno, 84081 Baronissi, Italy

We read with great interest the article by Naranjo-Bonilla et al. concerning changes in retinal and choroidal thickness (ChT) in patients with obstructive sleep apnea (OSA) who underwent continuous positive airway pressure treatment (CPAP) [1]. We really appreciated this paper, not only because OSA is a chronic respiratory disease that is linked to ocular and systemic disorders leading to an overall increased mortality risk, but also because ChT has been involved in several ocular diseases. Therefore, this topic is very interesting [2,3,4]. However, we have some concerns about the way this study was performed. 

In this study, the authors measured ChT on images on the pixel/microns scale using a line perpendicular to the choroid–sclera junction. To the best of our knowledge, ChT evaluation should be performed on images on the microns scale and perpendicularly to the RPE–Bruch complex [5].

In addition, we are a bit concerned about the measurement technique in their representative figures; the drawn lines in Figure 2/B of the RPE are not exactly at the RPE level and are not perpendicular to the choroid–sclera junction. We wonder why the nasal and temporal ChT measurements were performed at different distances from the fovea; the authors drew two different lines, one 1019 microns nasally and the other 1095 microns temporally to the fovea. 

Furthermore, to obtain reliable and comparable results in case of follow-up examinations, the images should be taken utilizing the follow-up setting. We wonder why the measurements obtained from the exams at 3 and 12 months were not executed with such a setting.

Even if we are aware that the measurements of axial length are not very precise [6], it has been clearly shown that ChT is related to the axial length: the choroid is thicker in shorter eyes and thinner in longer ones. Unfortunately, in this study, patients’ axial length was not evaluated, making the results questionable.

In addition, in this study, the results after the CPAP treatment were only compared to the baseline patient, without a control group of people affected by OSA but not treated with CPAP. Without a control group, how can the authors state that the results were caused by CPAP treatment and not by the disease itself?
